# Association between Polypharmacy and Hospitalization among Older Adults Admitted to Emergency Departments for Falls in Guadeloupe: A Retrospective Cohort Study

**DOI:** 10.3390/healthcare12151471

**Published:** 2024-07-24

**Authors:** Nadine Simo-Tabue, Laurys Letchimy, Ludwig Mounsamy, Leila Rinaldo, Larissa Vainqueur, Marie-Josiane Ntsama-Essomba, Guillaume Mallet, Denis Boucaud-Maitre, Maturín Tabue Teguo

**Affiliations:** 1 Unité de Recherche EpiCliV, Université des Antilles, 97233 Fort-de-France, France; 2 Service de Gériatrie, CHU de Martinique, 97261 Fort-de-France, France; 3 Pôle Gériatrie-Gérontologie, Université des Antilles, 97233 Fort-de-France, France; 4 CHU de Martinique, 97261 Fort-de-France, France; 5 CHU Pointe à Pitre, Université des Antilles, 97233 Fort-de-France, France; ludwig.mounsamy@chu-guadeloupe.fr (L.M.);; 6 Hôpital Central de Yaoundé, Yaoundé P.O. Box 25625, Cameroon; ebomaj2012@gmail.com; 7 Centre Hospitalier le Vinatier, 69500 Bron, France; 8 Epidémiologie Clinique et Vieillissement (EpiCliV), Université des Antilles, 97233 Fort-de-France, France; 9 Département de Recherche Clinique et Innovation, CHU de Martinique, 97261 Fort-de-France, France; 10 Equipe ACTIVE, Université de Bordeaux, 33405 Talence, France

**Keywords:** fall, elderly, Guadeloupe, emergency

## Abstract

**Introduction:** Falls are a common geriatric syndrome in older people. Falls are associated with adverse health events such as dependency, unplanned emergency admissions and death. This study aimed to identify the factors associated with fall severity, such as diabetes, hypertension, heart disease, cognitive decline and polypharmacy, as well as sociodemographic characteristics in patients aged 70 years and over admitted to the emergency department in Guadeloupe. **Method:** A single-center, observational, retrospective study of patients aged 70 years and over admitted to the emergency department (ED) of the University Hospital of Guadeloupe for a fall between 1 May 2018 and 30 April 2019 was conducted. Fall severity was defined as the need for hospitalization. Bivariate analysis was used to determine the associations between fall severity and sociodemographic characteristics, comorbidities, history of falls and polypharmacy (defined as the daily use of at least five drugs). Polypharmacy was analyzed as a binary variable (>5 drugs daily; yes or no) in categories (0–3 (ref.), 4–6, 7–9 and ≥10 drugs). **Results:** During the study period, 625 patients who attended the ED for a fall were included. The mean age was 82.6 ± 7.6 years, and 51.2% were women. Of these, 277 patients (44.3%) were admitted to the hospital, and 3 patients (0.5%) died. In the bivariate analysis, only polypharmacy was associated with hospitalization for a fall (OR: 1.63 [95% CI: 1.33–2.02]). The odds ratios for the polypharmacy categories were 1.46 [95% CI 0.99–2.14], 1.65 [1.09–2.50] and 1.48 [0.76–2.85] for 4–6, 7–9 and ≥10 drugs, respectively. **Conclusions:** Polypharmacy was associated with hospitalization as a proxy for fall severity. A regular review of drug prescriptions is essential to reduce polypharmacy in older adults.

## 1. Introduction

Falls are a common geriatric syndrome in older people [1,2]. A fall is defined as any involuntary event that causes the person to come to rest on the ground or at a lower level than the starting position without being the result of an acute incident such as a stroke [3]. Falls predict adverse health events, such as the need for admission to a nursing home, attendance at an emergency department (ED) and death [4]. The mortality rate attributable to falls among older people in the community is estimated at 5%, corresponding to around 9000 deaths per year among people aged 75 years and over in France. The annual risk of hospitalization due to falls is estimated to be 20% [5]. Falls are furthermore associated with a reduction in the quality of life [6,7,8], and are costly from a medico-economic perspective [9]. 

Several studies have focused on preventing falls in older people [10] in the community [11] or in nursing home [12] and hospital [13] settings. In a review of the literature on interventions to reduce falls in older people in hospital, Morris et al. showed that falls in hospitals can be prevented mainly by training healthcare professionals and raising their awareness [3]. Numerous interventions have been shown to be effective in preventing falls [14,15,16,17]. In a recent study carried out in Portugal in 2021, the authors investigated hospital admissions due to falls and found that they accounted for 2.1% of the total number of hospital admissions over a period of 9 years [18]. Vu et al. found a high prevalence of recurrent falls among older people hospitalized due to fall injuries in Vietnam [19]. Polymedication is a common geriatric syndrome among the elderly [20]. It is defined as “the simultaneous administration of many drugs or the administration of an excessive number of drugs”. It is estimated that between 14% and 49% of patients aged over 75 take more than five medications a day [21]. As with falls, it is linked to adverse health outcomes, including mortality, hospitalization and falls [22]. The risk of falling increases with each new treatment prescribed [23]. 

To the best of our knowledge, in the Caribbean region [24,25,26], and in Guadeloupe in particular [27], very few studies have investigated falls and fall severity in the region. The aim of this study was therefore to identify the factors associated with fall severity (as assessed by need for hospitalization) in patients aged 70 years and over attending the emergency department in Guadeloupe.

## 2. Methods

### 2.1. Study Design 

Setting: This was a retrospective, observational, single-center study of older adults in the emergency department of the University Hospital of Guadeloupe.

Setting: In this region of almost 380,000 inhabitants, there are three emergency departments (ED), one in the private sector and two in the public sector. The emergency department of the University Hospital of Guadeloupe accounts for 2/3 of all stays in the region. Therefore, only this ED participated in this study from 1 May 2018 to 30 April 2019 (i.e., prior to the start of the COVID-19 pandemic).

Participants: All patients aged 70 years and older who attended the ED because of a fall were identified from the hospital informatics database. The keywords used were “fall” (“fell/fallen”). 

Inclusion criteria: The inclusion criteria were as follows: age ≥ 70 years, fall as the reason for consultation and a completed entry prescription. 

Exclusion criteria: Patients were excluded from the study if their entry prescription was incomplete or could not be found.

Ethical issue: All patients admitted to the emergency department of the University Hospital Center were informed that their data may be used for research purposes. Informed consent was obtained from all subjects and/or their legal guardian(s). All participants and their representatives were informed about the study by the investigators and were given the opportunity to specifically opt out of the use of their medical data for research purposes. Those who expressed opposition were not recruited. The research was carried out using Resurgence* computer software (https://www.resurgencecomputers.com/home), which is used on a daily basis in the emergency department. In compliance with the Declaration of Helsinki and French data protection laws, the study protocol was approved by the Ethics Committee of the University Hospital of Guadeloupe on 25 August 2019. All methods were applied in accordance with applicable guidelines and regulations.

### 2.2. Outcome Measure

The outcome of interest was fall severity, defined as the need for hospital admission from the ED. 

### 2.3. Other Variables

Sociodemographic characteristics (age and sex); presence of chronic diseases and/or geriatric syndromes (hypertension, diabetes, cognitive disorders and history of falls in the past 12 months); functional status (dependence); place of residence (nursing home, at home or foster family); chronic/acute alcohol use and ongoing medication were also recorded. 

### 2.4. Statistical Analysis

Quantitative variables are expressed as the mean ± standard deviation and categorical variables as percentage. Ongoing medications were grouped by International Common Denomination and then counted. Dependency was classified into two categories: totally dependent versus not totally dependent. The polypharmacy variable was considered first in binary form (>5 drugs daily; yes or no) and in categories (0–3 (reference), 4–6, 7–9 and ≥10 drugs). Bivariate logistic regression analyses were performed to investigate the relationship between fall severity and each variable. *p*-values < 0.05 were considered statistically significant. All analyses were conducted using R.3.0.2.

## 3. Results

A total of 813 patients attended the ED of the CHU of Guadeloupe for falls during the study period. After the exclusion of duplicates and false positives, 658 patients were eligible for the study. For thirty-three people, we were unable to retrieve treatment orders. Finally, 625 patients were included in the study. The mean age of the participants was 82.6 ± 7.7 years, and 51.2% were women. In total, 277 patients (44.3%) required hospital admission, while the remaining 348 (55.7%) were discharged home. Three patients (0.5%) died in the ED after admission. For the outcomes of the remaining 274 patients, 181 (66.1%) were admitted to a medical ward and 93 (33.9%) were admitted to a surgical ward. 

A total of 248 patients (39.7%) had polypharmacy (>5 drugs daily). In total, 32.5% had diabetes, 66.9% had hypertension, 19.5% had cognitive decline, 7.5% consumed alcohol and 7.4% had severe dependency (Table 1). Thirty-three patients were living in a nursing home or foster families. A quarter of the patients had a history of at least two falls in the previous year. 

In the bivariate analyses, only polypharmacy was associated with fall severity (OR: 1.63 [95% CI: 1.33–2.02]) (Table 1). The other clinical characteristics were not associated with fall severity. When the number of drugs was considered in categories with 0–3 drugs as the reference, we observed that the categories 4–6 (OR: 1.46 [1.00–2.14]) and 7–9 drugs (OR: 1.65 [1.09–2.50]) were associated with fall severity, whereas the category ≥10 drugs (N = 42 patients) (OR: 1.48 [0.76–2.85]) was not (Table 2). The percentages of fall severity were 38.1%, 47.3%, 50.3% and 47.6% for 0–3 drugs, 4–6 drugs, 7–9 drugs and ≥10 drugs, respectively (Table 2).

## 4. Discussion

In this study, we observed that only polypharmacy (>5 drugs) was associated with hospital admission (OR: 1.63 [1.33–2.02]) in adults aged 70 years and over attending the ED of the University Hospital of Guadeloupe for a fall. This finding is consistent with a previous study by Morin et al. on a cohort of almost 50,000 patients, showing that there was an association between the number of medications prescribed and the risk of injurious falls [28]. In Morin’s study, polypharmacy was defined as the use of than four medications (number of medications taken in the last 7 days and sorted according to the Anatomical Therapeutic Chemical (ATC) classification). The authors showed a 2% increase in falls among people taking multiple medications. However, after comprehensive adjustment for known confounders (including fall risk-increasing drugs and chronic multimorbidity), this association was substantially weaker than previously reported. Moreover, even if the relationship between polypharmacy and injurious falls is really causal, the population attributable risk fraction is low [28]. A similar association was also found by Laflamme et al. in a case–control study [29]. They found an increased risk of fall injury after two treatments [29].

In our study, when categorizing the number of medications taken, using the group taking 0–3 drugs as the reference category, we observed an increased risk of fall severity among older adults taking 4–6 medications and 7–9 medications. Nevertheless, no association was observed in the case of excessive polypharmacy (≥10 drugs), contrary to the studies by Laflamme et al. and Morin et al., where the relationship persisted at high numbers of drugs [28,29]. This difference compared to our work may be related to the difference in the main assessment criteria (i.e., definitions of fall severity and polypharmacy), as well as the sample size. The relatively small number of overmedicated patients (N = 42) in our study may contribute to the discrepancy between our findings and those reported in the existing literature. Indeed, we observed a similar rate of hospital admission in this subgroup (47.6%) compared to the 4–6 (47.3%) and 7–9 (50.3%) subgroup categories. 

Further investigation is required to confirm these results. In this context, the administration of multiple drugs may be associated with therapeutic optimization strategies (in considering the quantitative and qualitative aspects of drugs) in this population, which are characterized by a high prevalence of underlying chronic pathologies. Patients with chronic conditions require a sufficient number of drugs to achieve therapeutic efficiency. Nevertheless, it is important to note that the risk of potential adverse health events remains high in patients receiving unnecessary treatments. It is therefore of the utmost importance that caution is exercised and that prescriptions are regularly reevaluated.

In our study, we did not find any association between cognitive impairment and fall severity. This finding is not consistent with the data in the literature, where it is well established that cognitive impairment is a risk factor for falls [24,30,31]. This lack of association may be partly due to the lack of power of the study or the lack of a precise diagnosis regarding cognitive impairment. Indeed, the data were retrieved from the patients’ medical records, and no cognitive testing was performed during the ED visits. It is important to note that the majority of studies use the occurrence of a fracture (especially fractures of the upper end of the femur) as a criterion to assess the severity of a fall. After this type of fracture, the 6-month mortality rate for patients with a Mini Mental State Examination (MMSE) score < 20 is estimated to be 50%, compared to 11% for those with a normal MMSE score [32]. The threshold used by Tinetti was a MMSE < 26 (OR: 2.8 [95% CI: 1.7–4.7]) [33]. A history of repeated falls (≥2 falls in the previous 12 months) was not significantly associated with fall severity in our study. Repeated falls are predictive of adverse health events according to the French National Health Agency. However, 95% of recurrent falls are classified as non-serious, i.e., without fracture, cranial trauma or major skin laceration [5]. Several studies have shown that recurrent falls (>2 per year) are predictive of adverse health events in older people. These include incident dependency, hospitalization and mortality. This means that recurrent falls can be considered as a prognostic marker and/or a marker of fall severity (depending on the definition). The definition of fall severity (hospitalization and non-trauma) and the small number of patients in our study may explain the lack of significance. It should also be noted that the measurement of the “fall” variable may have lacked precision. Indeed, the local informatics database software does not include “history of falls” as a variable to be recorded during the clinical examination of older people. This information was only provided verbally during history taking or by reviewing the history of visits due to falls. Acute or chronic alcohol consumption also did not appear to be associated with fall severity in our study, but this may be due to the small number of persons in this category. Alcohol use is well known to be a risk factor for falls [34,35]. 

To the best of our knowledge, our study is the first to address the problem of falls among older adults in Guadeloupe. From a clinical point of view, our study highlights the value of optimizing medical therapy and thus fall prevention in older people. Our findings may contribute, albeit modestly, to changing current practices and to raising awareness about the impact of medication on the severity of falls. It may also form the basis for more in-depth future studies. In addition to international studies, our findings will encourage practitioners to minimize prescriptions as much as possible in older people (qualitative reduction or the concept of therapeutic optimization). Some limitations of our study should be noted. Firstly, patients were selected using the search engine integrated into the “statistics” function of the database software. It is plausible that some patients with falls may not have been identified by this search function and that the final number retained is not fully representative of all patients with falls aged 70 years and over who had an emergency department visit during this period. Second, this was a retrospective study. Third, medical records in the ED are completed for information purposes and mostly verbally, leading to a potential loss of information. Finally, the University Hospital of Guadeloupe experienced a period of crisis following an accidental fire at the facility in November 2017. The number of inpatient beds was drastically reduced (by around 200 beds), and this may have impacted the outcomes of patients who suffered falls because of a lack of downstream beds.

## 5. Conclusions

Our study showed an association between only polymedication (no link was identified between excessive polymedication) and fall severity in patients aged 70 years and over-attending the emergency department in Guadeloupe. This result highlights the cooccurrence of these two geriatric syndromes, which are most often managed by the attending physician. Longitudinal studies with larger numbers of patients are needed to explore the relationship between excessive polymedication and fall severity.

## Figures and Tables

**Table 1:** Comparison of clinical characteristics of patients with and without hospitalization following a fall.

Variable	Overall N = 625	Admitted n = 277	Not Admitted n = 348	OR (95% CI)	*p*-Value
Age (years ± SD)	82.6 ± 7.7	82.4 ± 7.2	82.8 ± 8.1		0.522
Female, n (%)	320 (51.2%)	138 (49.8%)	182 (52.3%)		0.538
Institutionalized, n (%)	33 (5.3%)	14 (5.1%)	19 (5.5%)		0.821
Not totally dependent	579 (92.6%)	255 (92.1%)	324 (93.1%)		0.619
Diabetes	203 (32.5%)	90 (32.5%)	113 (32.5%)		0.996
Hypertension	418 (66.9%)	186 (67.2%)	232 (66.7%)		0.899
Heart disease	84 (13.4%)	42 (15.2%)	42 (12.1%)		0.260
Alcohol	47 (7.5%)	18 (6.5%)	29 (8.3%)		0.387
Stroke	81 (13.0%)	35 (12.6%)	46 (13.2%)		0.829
Epilepsy	34 (5.4%)	11 (4.0%)	23 (6.1%)		0.149
Parkinson	39 (6.2%)	16 (5.8%)	23 (6.6%)		0.669
Cognitive decline	122 (19.5%)	53 (19.1%)	69 (19.8%)		0.828
History of falls					
<2 in the previous year, n (%)	472 (75.5%)	210 (75.8%)	262 (75.3%)		0.879
≥2 in the previous per year, n (%)	153 (24.5%)	67 (24.2%)	86 (24.7%)		
Polypharmacy (>5 drugs), n (%)	248 (39.7%)	134 (48.4%)	114 (32.8%)	1.63 [1.33–2.02]	<0.001
Duration of stay in ED (hours ± SD)	10.4 ± 6.0	10.3 ± 6.0	10.5 ± 6.0		0.693

Notes: Results are presented as the means ± SDs or percentages. ED, emergency department, SD: standard deviation and CI: confidence interval.

**Table 2:** Association between the number of drugs and need for hospital admission.

Number of Drugs	Percentage of Hospital Admission	Odds Ratio (95% CI)	*p*-Value
0–3 (n = 252)	38.1%	Reference	
4–6 (n = 188)	47.3%	1.46 [1.00–2.14]	0.049
7–9 (n = 143)	50.3%	1.65 [1.09–2.50]	0.018
≥10 (n = 42)	47.6%	1.48 [0.76–2.85]	0.244

CI, confidence interval.

## Data Availability

All data generated or analyzed during this study are included in this published article. The datasets used and/or analyzed during the current study are available from the author Nadine Simo-Tabue upon reasonable request. The datasets generated and/or analyzed during the current study are not publicly available. The authors confirm that all experiments were performed in accordance with the relevant guidelines and regulations.
